# An X-ray harmonic separator for next-generation synchrotron X-ray sources and X-ray free-electron lasers

**DOI:** 10.1107/S160057751800108X

**Published:** 2018-02-28

**Authors:** Ichiro Inoue, Taito Osaka, Kenji Tamasaku, Haruhiko Ohashi, Hiroshi Yamazaki, Shunji Goto, Makina Yabashi

**Affiliations:** a RIKEN SPring-8 Center, 1-1-1 Kouto, Sayo, Hyogo 679-5148, Japan; b Japan Synchrotron Radiation Research Institute, 1-1-1 Kouto, Sayo, Hyogo 679-5198, Japan

**Keywords:** harmonic radiation, X-ray free-electron lasers, diffraction-limited storage rings, harmonic separation, SACLA

## Abstract

An X-ray prism for the extraction of a specific harmonic of undulator radiation is proposed. By using the prism in a grazing incidence geometry, the beam axes of fundamental and harmonics of undulator radiation are separated with large angles over 10 µrad, which enables the selection of a specific harmonic with the help of apertures, while keeping a high photon flux. The concept of the harmonic separation was experimentally confirmed using X-ray beams from the X-ray free-electron laser SACLA.

## Abstract 2

A new optical technique used to extract a specific harmonic of undulator radiation is proposed and the concept was experimentally confirmed using X-ray pulses from SACLA.

## 1. Introduction   

Continuous evolution of synchrotron radiation (SR) X-ray sources has strongly driven innovative sciences. The next-generation SR X-ray sources based on a multi-bend achromat lattice (Hettel, 2014 ▸; Einfeld *et al.*, 2014 ▸), sometimes called diffraction-limited storage rings, are currently emerging (Biasci *et al.*, 2014 ▸; Eriksson *et al.*, 2014 ▸; Yabashi & Tanaka, 2017 ▸). Compared with the present third-generation SR sources, the new SR sources produce X-ray beams from electron beams with much smaller emittances (the product of the spatial size and the angular spread of the particle/optical beam) closer to a natural emittance of X-ray light, which significantly enhances the brilliance of undulator radiation.

**Figure 1:**
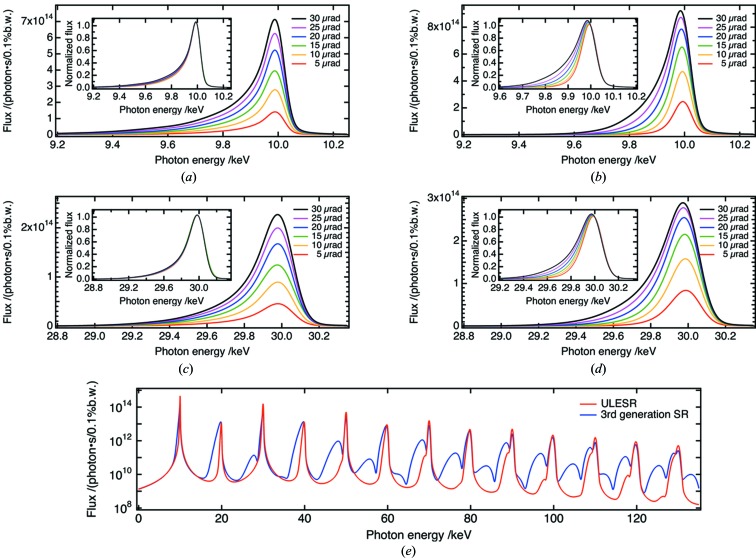
(*a*)–(*d*) Spectra of undulator radiation at typical third-generation SR and ULESR sources, simulated with parameters in Table 1 ▸ for a fixed vertical angular aperture size (10 µrad) and different horizontal aperture sizes (5, 10, 15, 20, 25, 30 µrad) at 30 m downstream of the source. (*a*) and (*b*) Spectra of fundamental undulator radiation at (*a*) the third-generation SR and (*b*) ULESR source. The insets show the spectra normalized to the flux at 10 keV. (*c*) and (*d*) Spectra of the third-harmonic undulator radiation at (*c*) the third-generation SR and (*d*) ULESR source. The insets show the spectra normalized to the flux at 30 keV. The undulator spectra at the third-generation source are almost the same shape for different conditions of the aperture size (inset of *a* and *c*). On the other hand, the shapes of undulator spectra at the ULESR source depend on the aperture size (*b* and *d*). For horizontal apertures less than 10 µrad, the spectral peaks are almost symmetric with a narrow bandwidth (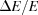 ≃ 1%) (*b* and *d*). (*e*) Comparison of the spectra of undulator radiation at the third-generation SR and ULESR sources simulated with the same parameters for an angular aperture size of 10 µrad (horizontal) 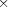 10 µrad (vertical).

Another interesting feature of these ultra-low-emittance SR (ULESR) sources is the symmetric spectral peak with a relative energy bandwidth 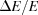 as small as ∼1% for each harmonic component of on-axis undulator radiation. This is in contrast to the wider spectral peaks (where 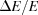 is about several percent) with broad tails to the lower photon-energy side at the present SR sources. Typical spectra of undulator radiation for different angular aperture sizes at a hypothetical third-generation SR and an ULESR that are calculated by the simulation code *SPECTRA* (Tanaka & Kitamura, 2001 ▸) are shown in Fig. 1 ▸ ▸. The symmetric spectral peaks at the ULESR sources come from the small electron-beam size that allows suppressing contamination of off-axis low-energy components into the central cone of undulator radiation with an angular divergence of ∼10 µrad.

In conventional SR sources with wider bandwidths, one has to utilize X-ray monochromators in most experiments. A double-crystal monochromator (DCM) with flat silicon (Si) crystals has been widely used as a basic optical component with a typical resolution 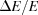 of 0.01% using the Si 111 reflection. The resolution can be smaller at higher photon energies, because higher reflection indexes with increased Bragg angles are sometimes preferable for geometrical reasons (*e.g.* 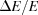 ≃ 0.005% with the Si 220 reflection). However, some applications require high-flux X-ray beams without excessive monochromatization. To realize high-flux X-ray beams, other X-ray monochromators are used. For example, multilayer monochromators (Chu *et al.*, 2002 ▸; Stampanoni *et al.*, 2007 ▸; Rack *et al.*, 2010 ▸) can increase the bandwidth up to a few percent, while fabrication of multilayers becomes technically more difficult at higher photon-energies, because one needs to realize shorter periodicity of multilayers with high accuracy. Alternatively, bent Laue monochromators (Shastri *et al.*, 2002 ▸) can be used to increase the bandwidth, while the bandwidth is limited up to 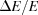 ≃ 0.1%.

In the ULESR sources, we are able to use extremely brilliant, pink X-ray beams with a moderate bandwidth of 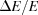 ≃ 1%, by extracting the central cone of a specific harmonic of undulator radiation and suppressing other harmonic components. This scheme will provide significant impact on various fields of applications, such as time-resolved pump–probe experiments and imaging techniques. In fact, the fundamental pink radiation (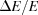 ≃ 0.5%) is widely used in XFEL applications, such as serial femtosecond crystallography (Chapman *et al.*, 2011 ▸; Schlichting, 2015 ▸), coherent diffraction imaging (Seibert *et al.*, 2011 ▸), solution scattering (Kim *et al.*, 2015 ▸), and X-ray diffraction of matter in extreme conditions (Milathianaki *et al.*, 2013 ▸). Moreover, this scheme can be used for extraction of harmonic XFEL radiation, which would enable us to perform new XFEL applications, as well as verify and develop XFEL theories for harmonic radiation (Huang & Kim, 2000 ▸, 2007 ▸; Saldin *et al.*, 2006 ▸; Pellegrini *et al.*, 2016 ▸).

One can simply use an X-ray reflective mirror to transport only the fundamental radiation while rejecting higher-order harmonics. However, extracting a specific harmonic component is not straightforward. In this paper, we present a simple combination of reflective and refractive optics, which we call a harmonic separator, to extract a specific harmonic of undulator radiation.

This paper consists of four sections. In §2, we describe the principle of the harmonic separation. §3 addresses the results of a proof-of-principle experiment of harmonic separation using self-amplified spontaneous emission (SASE)-XFEL beams at SACLA (Ishikawa, 2012 ▸). In §4, we provide a summary and future perspectives.

## 2. Principles   

**Figure 2:**
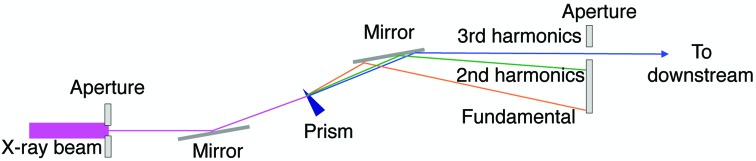
Schematic illustration of harmonic separation using an X-ray prism.

The schematic in Fig. 2 ▸ shows the principle method used to separate the third-harmonic radiation by an X-ray prism. The same scheme can be used for other harmonics. First, an entrance aperture selects the central cone of undulator radiation. A pair of cut-off mirrors remove the unwanted higher-order harmonic radiation. By inserting a wedge prism between the mirrors, fundamental and harmonic radiation are deflected at slightly different angles because of the dispersion effect. If the angular separation is larger than the beam divergence, we can extract a specific harmonic radiation simply by setting an exit aperture.

Since the angular divergence of the central cone at an ULESR source is typically 10 µrad, the differences in deflection angles of the prism should be larger than 10 µrad. We note that the differences around several microradians are enough for the XFEL light, because the angular divergence of the XFEL is a few microradians.

**Figure 3:**
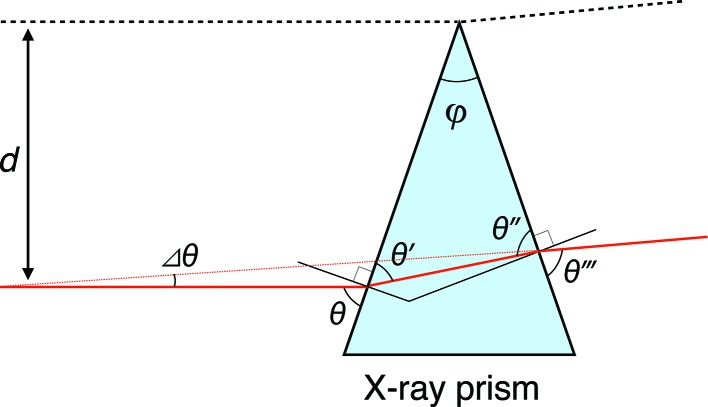
Schematic illustration of an X-ray prism and trajectory of the deflected X-ray beam.

Let us consider the situation where the monochromatic X-ray beam is incident on the prism with an apex angle φ and an incident angle 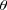, as shown in Fig. 3 ▸. We first assume that the absorption of the X-ray beam by the prism is negligible and that the index of refraction for the prism is given by 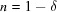. The following two equations relating to 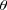 and the angles 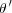, 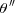, 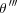 (defined in Fig. 3 ▸) hold from Snell’s law:
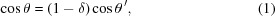


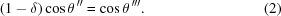
Since 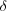 is of the order of 10^−6^ in the X-ray region, 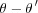 and 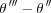 are much less than unity, equations (1) and (2) can be rewritten as
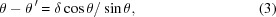


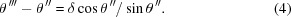
From the above two equations and the relation 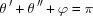, the deflection angle by the prism 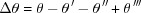 is given by
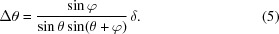
In a grazing incidence geometry, equation (5) can be rewritten as
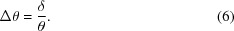
According to equation (6), one can increase the deflection angle by decreasing the incident angle 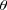. Here we emphasize that the deflection angle can be made constant for a wide range of photon energies by simply changing the incident angle, indicating the fixed-exit geometry can be easily realized.

Next, let us consider the situation where fundamental and harmonic undulator radiation irradiate the prism. If the X-ray photon energy is far from the absorption edges of the materials used in the prism, 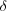 is almost inversely proportional to the square of the X-ray photon energy (Als-Nielsen & McMorrow, 2011 ▸). Thus, the difference in the deflection angle for *n*th and (*n*−1)th harmonic radiation in the grazing incidence geometry is approximated by
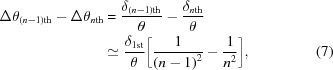
where 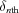 is 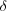 for the *n*th harmonic radiation. The incident angle can be reduced down to the critical angle for total reflection, which is of the order of millradians. Considering 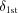 to be of the order of 10^−6^, we can make the angular deviation 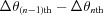 larger than the angular divergence of the undulator radiation (∼10 µrad) for practical numbers of *n* (*n* = 2–9).

For practical usage of the X-ray prism, the absorption by the prism should be considered. The transmittance is dependent on the irradiation position of the beam. Let us consider the transmission of a part of the incident X-ray beam (red line in Fig. 3 ▸) that is separated by a distance *d* to the straight line that penetrates the apex of the prism and is parallel to the incident X-ray beam. In this case, the X-ray beam passes the prism by a distance 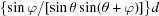, and thus the transmittance is given by 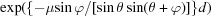 with the linear absorption coefficient 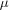. Assuming that the shape of the incident beam is a flat-top with width *w* and one edge of the beam irradiates the apex of the prism, the average transmittance of the X-ray beam 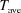 can be expressed as
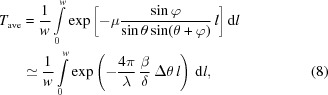
where 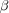 is the imaginary part of the index of refraction of the prism and λ is the wavelength. Also, the transmittance at the edge of the beam opposite to the apex of the prism 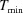 is given by 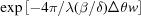. Generally, 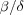 is smaller for lighter elements. Thus, prisms made of light elements are suitable for achieving large deflection angles and high throughput.

To investigate the feasibility of the harmonic separation, we consider prisms made out of glassy carbon with a density 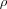 = 1.51 g cm^−3^, diamond (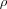 = 3.52 g cm^−3^), silicon (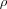 = 2.33 g cm^−3^) and germanium (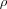 = 5.33 g cm^−3^) with an apex angle of π/2. Here we discuss two situations. We assume that the incident beam has a top-hat shape with a width *w* of 300 µm, which corresponds to the profile of the X-ray beam with an angular divergence of 10 µrad at 30 m downstream of the light source. The prism is positioned such that it fully intercepts the beam with one beam edge coinciding with the prism apex. The first case is an extraction of the third harmonic (30 keV) of undulator radiation with a fundamental photon energy of 10 keV. Table 2 ▸ shows the incident angles of the respective prisms and related parameters for achieving 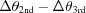 to be 10 µrad. Also, 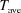 and 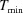 for the third harmonic are shown. As shown in this table, the angular separation of the second and third harmonics can be readily achieved in all cases of the prism, indicating that one can extract the third harmonic by setting an aperture downstream of the prism. Also, we note that high throughput greater than 80% can be realized by using prisms made from carbon, while the throughput of the other prisms in Table 2 ▸ is much lower than that of glassy carbon and diamond prisms.

The second is an extraction of the fifth harmonic (70 keV) from undulator radiation with a fundamental photon energy of 14 keV. Table 3 ▸ shows the incident angles of the respective prisms and related parameters for achieving 10 µrad for 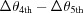. 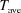 and 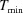 for the fifth harmonic are also shown. Although the incident angle should be much smaller with respect to the previous case as a result of the small difference between 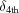 and 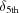, we can readily achieve the angular separation of the fourth- and fifth-harmonic radiation. Also, reasonably high throughput of the extracted undulator radiation (over 40%) can be realized by using glassy carbon or diamond prism, while the throughput of the other prisms is much lower than that of the prisms made from carbon.

Finally, we discuss the dispersion effect caused by the prism. Due to the finite energy spread of each harmonic radiation, extra divergence arises when the X-ray beam transmits the prism. Since 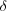 is almost inversely proportional to the square of the X-ray photon energy, the additional divergence is approximated to be 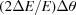, which is much smaller than the deflection angle 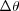 and the angular divergence of the undulator radiation. Therefore, the dispersion effect is negligible in the present scheme.

## 3. Experimental   

We performed a proof-of-principle experiment of harmonic separation using the XFEL light source SACLA (Ishikawa, 2012 ▸) with a fundamental photon energy of 10 keV. We used an X-ray prism with an apex angle of 90° made out of glassy carbon (Tokai carbon). The prism surfaces were mechanically polished. The density of the glassy carbon was 1.51 g cm^−3^, which corresponds to 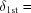 3.14 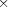 10^−6^, 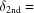 7.83 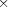 10^−7^ and 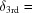 3.48 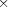 10^−7^.

**Figure 4:**
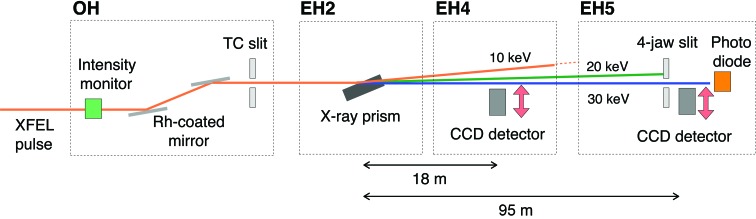
Schematic illustration of experimental setup for harmonic separation at SACLA BL3.

Fig. 4 ▸ shows the side view of the experimental setup. We operated SACLA BL3 (Tono *et al.*, 2013 ▸; Yabashi *et al.*, 2015 ▸) to generate 10 keV fundamental XFEL light at a repetition rate of 30 Hz. The Rh-coated X-ray mirrors in the optical hutch (OH) removed harmonic X-ray radiation above the fourth order. We positioned the X-ray prism in experimental hutch 2 (EH2) in the grazing incidence geom­etry and the X-ray beam was deflected in the vertical direction. The intensity profiles of the deflected XFEL beams were measured at two positions, 18 m (EH4) and 95 m downstream (EH5) from the prism, with two charge-coupled device (CCD) detectors (Hamamatsu Photonics ORCA-R2 in EH4 and Hamamatsu Photonics ORCA-Flash in EH5). We also utilized a four-jaw slit and a photodiode in EH5 for monitoring intensities of harmonic radiation in a single-shot manner.

**Figure 5:**
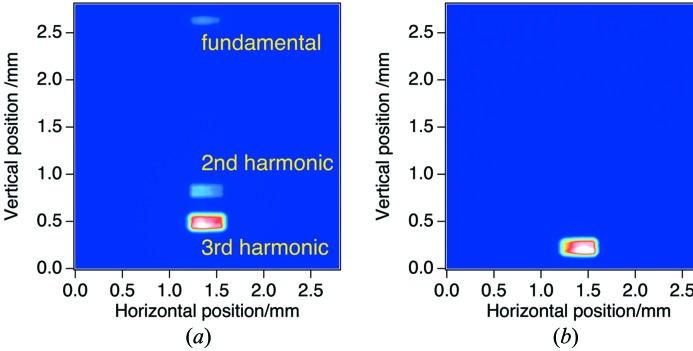
XFEL beam profiles in EH4 transmitted through the prism (*a*) and without prism (*b*).

First, we tested the angular separation of the fundamental, second and third harmonics of the XFEL light. We selected a central part of the XFEL beam by transport channel (TC)-slit in OH with an aperture size of 310 µm (H) 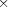 150 µm (V). The incident angle 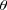 was set at 1.3°, and the spatial profile of the XFEL beam that transmitted the prism was measured. Fig. 5 ▸(*a*) shows the beam profile measured by the CCD detector in EH4 with an exposure time of 500 ms, which corresponds to 15 XFEL pulses. For reference, an XFEL beam profile without the prism is also shown in Fig. 5 ▸(*b*). Here the silicon (Si) attenuator in OH with an appropriate thickness is inserted to make the intensities of the fundamental and harmonic radiation comparable. As shown in the figure, XFEL beams were split into three beams after transmission of the prism. The displacements of the deflected fundamental, second and third harmonics with respect to the beam position without the prism were 2.4, 0.60 and 0.26 mm, respectively. These values correspond to the deflection angles of 133 µrad, 33.3 µrad and 14.4 µrad, respectively, which are in reasonable agreement with the theoretical values calculated by equation (5) (138, 34.5 and 15.3 µrad for 10, 20 and 30 keV X-ray beams, respectively). We note that the beam profile does not contain any speckle at the spatial resolution of the detector (∼10 µm).

Next, we demonstrate that the X-ray prism can be used for photon diagnostics of the harmonic XFELs. We measured the intensity growth of the harmonic XFEL radiation as a function of undulator length *L* (*i.e.* gain curve). Under the normal operating conditions of SACLA to generate 10 keV fundamental XFEL pulses, we changed the number of operating undulators by opening the gap of undulators one-by-one from downstream to upstream. We opened the TC slit to have the dimensions 1900 µm (H) 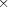 550 µm (V), which are values larger than the beam size of the fundamental radiation. We set the X-ray prism in EH2 with an incident angle of 2° and spatially separate the second and third harmonics in EH5. Here we note that the fundamental radiation did not reach EH5 because of the limited beamline aperture. By extracting either of the second or third harmonics using a four-jaw slit in EH5, we measured the pulse energy of the XFEL beam that transmitted the prism in a single shot manner by a photodiode located downstream of the slit.

**Figure 6:**
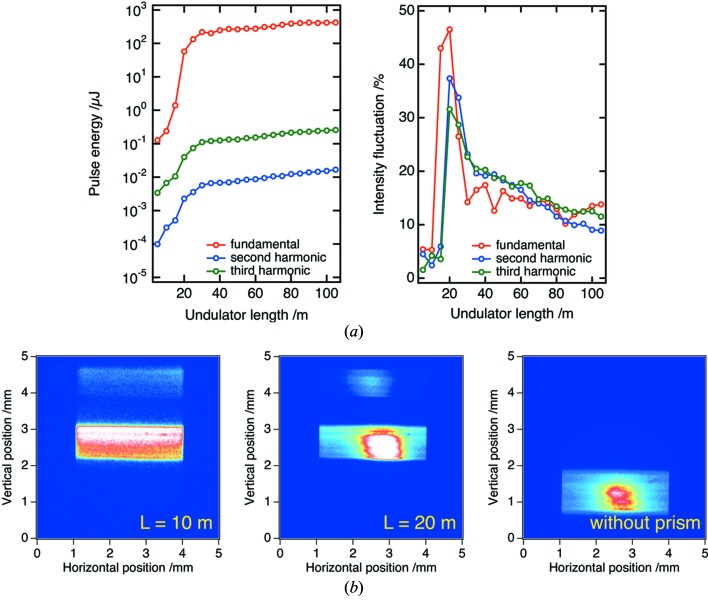
(*a*) Average pulse energies and intensity fluctuations as a function of undulator length for the second and third harmonics of the XFEL beam that transmitted the prism and those for fundamental radiation in OH. (*b*) XFEL beam profiles in EH5 that transmitted the prism [(left) *L* = 10 m, (middle) *L* = 20 m] and without the prism [*L* = 105 m] (right).

Fig. 6 ▸(*a*) is the average pulse energy of the second- and third-harmonic radiation with that of the fundamental radiation measured by the intensity monitor in OH as a function of *L*. We also show the standard deviations of the pulse energies (intensity fluctuations) of the fundamental and harmonic radiation. The pulse energy of harmonic radiation gradually increased as *L* increased from *L* = 5 m, then suddenly grew exponentially at *L* ≃ 20 m. Finally, the pulse energy of harmonic radiation saturated at *L* ≃ 50 m. The exponential intensity growth is a clear sign for the transition of harmonic radiation from spontaneous radiation to laser light. In fact, the beam profiles of the harmonic radiation were greatly different for the cases before and during this steep intensity growth. Fig. 6 ▸(*b*) shows the XFEL beam profile for the cases of *L* = 5 m and *L* = 20 m, which were measured by the CCD detector in EH5 with an exposure time of 4 s. Also, the beam profile without the prism (*L* = 105 m) is shown for reference. The harmonic radiation in the exponential gain region consisted of SASE-XFEL with a narrow divergence angle (a few microradians) and the spontaneous radiation with a broad divergence angle, though we did not see such a small divergent beam for small *L*. The small intensity fluctuation of the harmonic radiation was maintained (∼5%) before the exponential growth regime, then increased to ∼30% in the exponential growth regime, and finally decreased to ∼15% in the saturation regime, as shown in Fig. 6 ▸(*a*). These phenomena are typical behaviours predicted by SASE-FEL theory for harmonic radiation (Saldin *et al.*, 2006 ▸; Huang & Kim, 2007 ▸). Interestingly, we found that the intensity fluctuation of fundamental radiation at *L* = 15 m was considerably large (∼50%), while those of harmonics remained small (∼5%). This result indicates that the transition from spontaneous radiation to the XFEL light of fundamental radiation takes place at shorter undulator length, compared with harmonic radiation. We should emphasize that this kind of diagnostic for harmonic radiation was initially made possible with the harmonic separator.

## 4. Summary and future perspectives   

In this paper, we proposed an X-ray prism optics method for harmonic separation of undulator radiation from ULESR sources and XFELs. The proof-of-principle experiment was performed using XFEL beams with the fundamental photon energy of 10 keV. We successfully separated the fundamental radiation, the second and the third harmonics without degradation of the beam profiles. Furthermore, we measured the gain curves of harmonic radiation using the prism.

The harmonic separation enables us to use high-energy X-ray beams with a much larger number of photons that is two orders of magnitude larger than that of a monochromatized beam generated with conventional DCMs. Such a dramatic increase of X-ray intensity will be beneficial for various kinds of experiments at future ULESR sources and the present XFEL facilities. Also, photon diagnostics of harmonic radiation of XFELs would be an intriguing subject for verification of FEL theory and the development of advanced schemes of XFEL generation, such as harmonic lasing (Schneidmiller & Yurkov, 2012 ▸).

An important challenge of the harmonic separation is the improvement of the transmission of the prism. One of the straightforward ways is to make a prism out of a light element, as discussed in §2. Beryllium (Be) would be a good candidate material to achieve higher transmission; it is widely used for speckle-free X-ray windows in current synchrotron sources (Goto *et al.*, 2007 ▸, 2011 ▸; Yabashi *et al.*, 2014 ▸), and thus fabrication of high-quality Be prisms would be feasible. Also, kinoform-shape prisms (Aristov *et al.*, 2000 ▸) will improve the transmittance. Another challenge for the harmonic separation is the extraction of harmonic undulator radiation above 100 keV. For this purpose, the stacking of multiple prisms would be a solution.

## Figures and Tables

**Table 1:** Parameters used for calculations of undulator spectra in Fig. 1 ▸ Parameters of the electron beam for the third-generation SR source are based on those of SPring-8 except the beam energy, while those for the ULESR source are based on the conceptual design report of SPring-8-II (SPring-8 II Conceptual Design Report, http://rsc.riken.jp/eng/pdf/SPring-8-II.pdf).

Parameters	Third-generation SR	ULESR
Electron beam		
Energy (GeV)	6	6
Average current (mA)	100	100
Circumference (m)	1435.95	1435.95
Natural emittance (nm rad)	2.4	0.15
Coupling constant	0.002	0.1
Energy spread	0.001	0.001
Betatron function (m)	31.2 (H), 5.0 (V)	5.5 (H), 3.0 (V)
		
Undulator		
Periodic length (mm)	18	18
Number of periods	200	200
Total length (m)	3.6	3.6
*K* value	1.33926	1.33926
Fundamental photon energy (keV)	10.0	10.0

**Table 2:** Deflection angles and transmittance of undulator radiation with a fundamental photon energy of 10 keV for prisms made out of glassy carbon, diamond, silicon and germanium

	Glassy carbon	Diamond	Silicon	Germanium
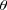 (mrad)	43.5	102.6	67.3	138.0
φ (rad)	π/2	π/2	π/2	π/2
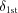	3.14 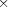 10^−6^	7.32 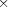 10^−6^	4.89 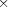 10^−6^	9.13 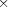 10^−6^
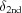	7.83 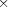 10^−7^	1.83 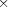 10^−6^	1.21 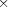 10^−6^	2.46 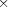 10^−6^
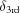	3.48 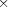 10^−7^	8.11 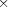 10^−7^	5.37 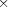 10^−7^	1.09 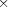 10^−6^
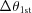 (µrad)	71.8	71.8	80.0	67.0
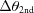 (µrad)	18.0	18.0	18.0	18.0
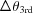 (µrad)	8.0	8.0	8.0	8.0
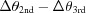 (µrad)	10.0	10.0	10.0	10.0
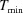 for 30 keV (%)(beam width: 300 µm)	79.8	79.8	26.1	1.1 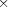 10^−5^
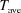 for 30 keV (%)(beam width: 300 µm)	89.5	89.5	55.0	6.2

**Table 3:** Deflection angles and transmittance of undulator radiation with a fundamental photon energy of 14 keV for prisms made out of glassy carbon, diamond, silicon and germanium

	Glassy carbon	Diamond	Silicon	Germanium
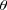 (mrad)	3.6	8.3	5.4	11.3
φ (rad)	π/2	π/2	π/2	π/2
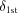	1.60 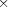 10^−6^	3.73 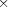 10^−6^	2.48 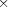 10^−6^	4.91 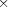 10^−6^
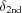	3.99 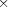 10^−7^	9.35 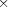 10^−7^	6.17 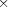 10^−7^	1.25 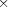 10^−6^
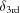	1.80 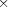 10^−7^	4.20 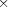 10^−7^	2.76 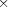 10^−7^	5.58 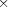 10^−7^
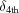	1.03 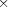 10^−7^	2.41 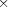 10^−7^	1.58 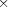 10^−7^	3.14 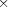 10^−7^
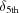	6.77 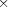 10^−7^	1.58 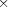 10^−7^	1.04 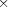 10^−7^	2.01 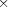 10^−7^
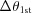 (µrad)	449.4	449.4	455.7	434.6
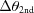 (µrad)	112.7	112.7	113.4	110.6
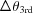 (µrad)	50.6	50.6	50.7	49.4
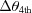 (µrad)	29.0	29.0	29.0	27.8
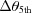 (µrad)	19.0	19.0	19.0	17.8
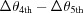 (µrad)	10.0	10.0	10.0	10.0
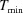 for 70 keV (%)(beam width: 300 µm)	11.8	11.8	3.6	7.4 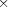 10^−7^
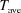 for 70 keV (%)(beam width: 300 µm)	41.2	41.2	29.0	5.3
